# Establishment and application of a triplex real-time PCR assay for detection of porcine circoviruses

**DOI:** 10.3389/fvets.2025.1558389

**Published:** 2025-03-03

**Authors:** Ze Cheng, Zenglin Wang, Lizhu Quan, Zhendong Zhang, Wenqiang Wang, Wei Wen, Zhenbang Zhu, Xiangdong Li

**Affiliations:** ^1^Jiangsu Co-innovation Center for Prevention and Control of Important Animal Infectious Diseases and Zoonoses, College of Veterinary Medicine, Yangzhou University, Yangzhou, China; ^2^Joint International Research Laboratory of Agriculture and Agri-Product Safety, The Ministry of Education of China, Yangzhou University, Yangzhou, China

**Keywords:** porcine circoviruses, triplex real-time PCR, epidemiological investigation, differential diagnosis, Anhui Province (China)

## Abstract

Porcine circovirus disease represents a prevalent ailment that is principally manifested by a series of clinical symptoms, including reproductive disorders in sows and high mortality rates in piglets. It has brought huge economic losses upon the global swine industry. Furthermore, the symptoms triggered by different genotypes of porcine circovirus bear resemblance and difficult to distinguish. Therefore, it is essential to establish a rapid, accurate, time-efficient, and high-throughput triplex real-time PCR differential diagnosis assay for detecting PCV2, PCV3, and PCV4. In this study, specific primers and probes were designed based on the conserved sequences of ORF1 sequences of PCV2, PCV3, and PCV4. The triplex Real-Time PCR assay was established and optimized, which showed satisfactory specificity, sensitivity, repeatability, and reproducibility. The limit of detection (LOD) was determined to 4.8×10^1^ copies/μL. The correlation coefficients R^2^ exceeded 0.999, and no cross-infection was found with other porcine viral pathogens. In addition, both the intra-repeatability and inter-repeatability were lower than 2%, which further attests to the reliability and stability of this assay. The complete consistency of the detection results with those of the commercial single-plex real-time PCR kits indicates that the established assay has satisfactory accuracy. The established assay was next applied to detect 370 clinical samples that were collected from 2023 to 2024 in the northern Anhui province of China. The results showed that the positive rate of PCV2 was 81.35% (301/370), the positive rate of PCV3 was 72.43% (268/370), and the positive rate of co-infection of PCV2 and PCV3 was 38.37% (142/370). However, PCV4 was not detected. Therefore, the established triplex real-time PCR assay in this study provides a valuable tool for the detection of porcine circovirus, which facilitates the epidemiological investigation of porcine circovirus in China.

## 1 Introduction

Porcine circoviruses (PCVs), which possess a circular, non-enveloped structure with single-stranded DNA, belong to the genus Circovirus within the family *Circoviridae* (1). Notably, they are currently recognized as the smallest known circular DNA viruses (2). At present, porcine circovirus encompasses four genotypes, specifically PCV1, PCV2, PCV3, and PCV4 (3). Except for PCV1, which is non-pathogenic, the remaining three porcine circoviruses are all capable of inducing comparable symptoms such as reproductive disorders, respiratory issues, and dermatitis (4, 5). Porcine circovirus has a propensity to trigger mixed infections in combination with other pathogens (6). It has been reported that co-infections of PCV2 and PCV3 occur frequently, causing huge economic losses to the pig industry (7, 8). Therefore, establishing a rapidly differential diagnosis is of crucial significance for the prevention and control of PCV-related diseases. Currently, there are various detection methods for porcine circovirus, including enzyme-linked immunosorbent assay (9, 10), colloidal gold immunochromatographic assay (11), conventional PCR (12), but the disadvantage of these methods are time-consuming and labor-intensive. Chen et al. (13) established a single real-time fluorescence quantitative PCR method capable of detecting PCV3. However, the single real-time PCR assay takes a long duration and fails to accurately identify the pathogens. Li et al. (14) established a duplex real-time PCR assay capable of simultaneously detecting PCV2 and PCV3. Sun et al. (15) established a duplex real-time recombinase-aided amplification assay for the simultaneous and rapid detection of PCV3 and PCV4. However, the duplex real-time PCR assay may overlook the detection of a certain genotype. Therefore, it is necessary to establish an assay that can conduct differential diagnosis on pathogenic porcine circoviruses (PCVs), accurately identifying the pathogens and providing a theoretical foundation for subsequent prevention and control measures. For this purpose, a triplex real-time PCR assay specific for PCV2, PCV3, and PCV4 was established and optimized in this study, which has the advantages of satisfactory specificity, high sensitivity, as well as excellent repeatability and reproducibility.

## 2 Materials and methods

### 2.1 Viruses and clinical samples

All viral DNA samples, used in this study were stored in our laboratory at −20°C until use, including the DNA samples of PCV2, PCV3, PCV4, Porcine Parvovirus (PPV), Pseudorabies virus (PRV), African Swine Fever virus (ASFV) and cDNA samples of Porcine Epidemic Diarrhea virus (PEDV), Porcine Deltacoronavirus (PDCoV), Porcine Rotavirus (PoRV), Porcine Reproductive and Respiratory Syndrome virus (PRRSV) and Classical Swine Fever virus (CSFV) (16, 17). As for the cDNA of above-mentioned RNA viruses’ preparation, the viral supernatants were used to extract RNA using TRNzol Universal Reagent (DP424) (TIANGEN BIOTECH, Beijing, China) and reverse transcribe into first-strand cDNA using HiScript III 1st Strand cDNA Synthesis Kit (+gDNA wiper) (Vazyme, Nanjing, China).

A total of 370 lung and lymph node samples suspected of being infected with porcine circovirus (PCV) were collected between October 2023 and February 2024 in the northern Anhui province of China.

### 2.2 Viral nucleic acid extraction

Take 0.5 g of each tissue samples and add 1 mL of PBS with grinding beads to each tube for homogenization. A Tissuelyser (Jingxin, Shanghai, China) was utilized to pulverize the sample, followed by centrifugation at 8000 rpm for 10 min at 4°C. The supernatant was employed to extract DNA using a Simply P Virus DNA/RNA Extraction Kit (Bioer Technology, Hangzhou, China) according to the manufacturer’s instructions. The DNA products were stored at −20°C until use.

### 2.3 Design of primers and probes

DNASTAR software (DNASTAR Inc., Madison, WI, USA) was used to confirm the unique and highly conserved regions within the rep gene in the PCV2, PCV3, and PCV4 genomes. Primer Express 3.0 software (Applied Biosystems, Foster City, CA, USA) was then used to design the primers and probes. At least 20 genome sequences for PCV2, PCV3, and PCV4 were downloaded from NCBI for comparison and analysis. The TaqMan probes for PCV2, PCV3, and PCV4 were labeled with FAM, ROX, and HEX at the 5′ ends, respectively. The quencher at the 3′ end of PCV2, PCV3, and PCV4 were MGB. The primers and probes were synthesized by Sangon (Shanghai, China).

### 2.4 Establishment and optimization of the triplex real-time PCR

The triplex real-time PCR assay involves three pairs of primers and three TaqMan specific probes for PCV2-PCV4. The concentrations of primers and probes were optimized as previously described (16). After optimization, a 20 μL reaction mixture system includes 10 μL 2 × 5G qPCR premix with UB (Toroivd, Shanghai, China), 1 μL of each pair of primers (10 μM), 0.25 μL of each pair of probes (10 μM), 2 μL of viral DNA and 1.25 μL of ddH_2_O. The triplex real-time PCR amplification was performed on the applied biosystems QuantStudio3 (Thermo Fisher Scientific, USA); the amplification condition was set at 37°C for 2 min, 95°C for 1 min, followed by 40 cycles of 95°C for 5 s and 60°C for 30 s.

### 2.5 Standard curve generation of the triplex real-time PCR

The amplified gene fragments of PCV2, PCV3, and PCV4 were linked together for synthesis and were subsequently cloned into the pUC57 vector by Genewiz (Suzhou, China). The plasmid was subjected to 10-fold serial dilutions (4.8 × 10^7^–4.8 × 10^1^ copies/μL) and then used as samples for establishing the standard curve of the triplex real-time PCR assay. The concentration of the plasmid was converted to the copy number using the following formula: y(copies/μL) = (6.02 × 10^23^) × (x(ng/μL) × 10^−9^DNA)/(DNA length×660) (18).

### 2.6 Specificity and sensitivity of the triplex real-time PCR

For the purpose of evaluating the specificity of the established triplex real-time PCR assay, DNA samples of PPV, PRV, ASFV and cDNA samples of CSFV, PRRSV, PEDV, PDCoV, PoRV were employed. The standard plasmid with a 10-fold serial dilution ranging from 4.8 × 10^7^ copies/μL to 4.8 × 10^1^ copies/μL were used as the templates to detect the sensitivity and the limit of detection (LOD) of the established triplex real-time PCR assay.

### 2.7 Repeatability and reproducibility of the triplex real-time PCR

The repeatability (intra-assay variability) and reproducibility (inter-assay variability) of the triplex real-time PCR assay were evaluated using three concentrations of plasmids (4.8 × 10^7^, 4.8 × 10^5^, 4.8 × 10^3^ copies/μL). For the evaluation of intra-batch variability, the plasmids of each above dilutions were repeatedly detected three times in 1 day by the same operator. For the inter-assay variability, each dilution was tested in six independent experiments by two operators at different times according to the MIQE guidelines (19). Finally, the coefficient of variation of Ct values was calculated based on the experimental results of both intra-batch and inter-batch.

### 2.8 Comparison of the triplex real-time PCR with the commercial single-plex PCR kits

Fifteen DNA samples with clear background were used to compare the established triplex real-time PCR in this study and commercial single-plex PCV2 or PCV3 real-time PCR kit (Unilevel, Shanghai, China). Five DNA samples were extracted from pigs infected PCV2, another five DNA samples were extracted from pigs infected PCV3, and last five DNA samples were extracted from healthy SPF pigs.

### 2.9 Clinical application of the triplex real-time PCR

A total of 370 lung and lymph node samples, which were suspected of being infected with PCV, were collected in northern Anhui province of China from 2023 to 2024. These samples were used to investigate the prevalence of PCV2, PCV3, and PCV4 using the established triplex real-time PCR assay.

## 3 Results

### 3.1 Primers and probes design and concentration optimization

At least 20 reference sequences of PCV2, PCV3, and PCV4 were, respectively, downloaded from NCBI. Primers and probes were designed for the most conserved gene sequences of the three viruses (Supplementary Figure S1). The 5′ ends of the probes used in this study were labeled with different fluorophores to ensure that there is no interference among the fluorescent signals of different viruses. The sequences of the primers and probes are listed in Table 1. The concentrations of primers and probes were optimized as described in the Materials and Methods section. The same reaction parameters were used throughout the study.

**Table 1:** Primers and probes used in this study.

Viruses	Primers and probes	Sequence (5′–3′)	Position	Genes	Amplicons
PCV2	PCV2-F	TACCAGCAATCAGACCCCGT	809–828	ORF1	118 bp
	PCV2-R	CGTGGATTGTTCTGTAGCATTCTT	903–926		
	PCV2-P	FAM-GTCCCAGCTGTAGAAG-MGB	852–867		
PCV3	PCV3-F	AGGGAAAGCCCGAAACACA	229–247	ORF1	133 bp
	PCV3-R	TACCGCTTTTTCCAACCTCTTT	340–361		
	PCV3-P	ROX-GAGTGGGAATCTATTGTGGAGTGTG-MGB	283–307		
	PCV4-F	GAAGTAGGAGAGCCCAGTGCC	372–392	ORF1	114 bp
PCV4	PCV4-R	AAATACAGACGGGAACTCACGG	464–485		
	PCV4-P	HEX-GACCTTAAAGCGGCTGTGGC-MGB	407–426		

F, R, and P indicate forward primer, reverse primer, and probe, respectively.

### 3.2 Establishment of the standard curve for the triplex real-time PCR assay

**Figure 1:**
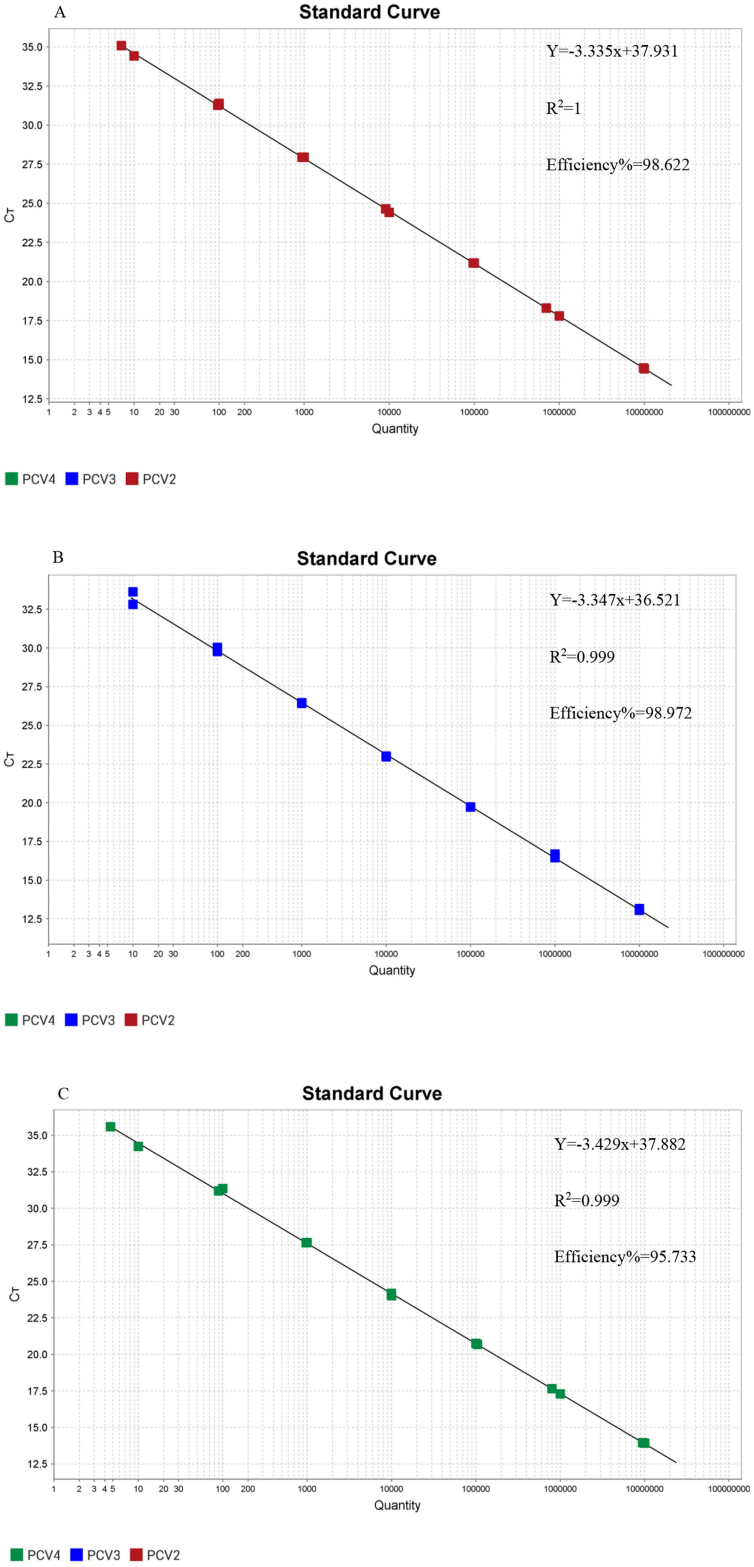
Standard curves of the triplex real-time PCR assay. **(A)** The standard curve for porcine circovirus type 2 (PCV2); **(B)** The standard curve for porcine circovirus type 3 (PCV3); **(C)** The standard curve for porcine circovirus type 4 (PCV4).

The standard curves of the triplex real-time PCR were constructed by using the constructed plasmid with a 10-fold serial dilution at concentrations ranging from 4.8 × 10^7^ copies/μL to 4.8 × 10^1^ copies/μL. As shown in Figure 1, the slopes of the standard curve for PCV2, PCV3, and PCV4 were −3.335, −3.347, and −3.429, respectively. The correlation coefficients R^2^ of PCV2, PCV3, and PCV4 were 1, 0.999, and 0.999, respectively. The amplification efficiency of PCV2, PCV3 and PCV4 were 98.622, 98.972, and 95.733, respectively. The above data proved that the triplex real-time PCR assay in this study had a high detection efficiency.

### 3.3 Specificity of the triplex real-time PCR

**Figure 2:**
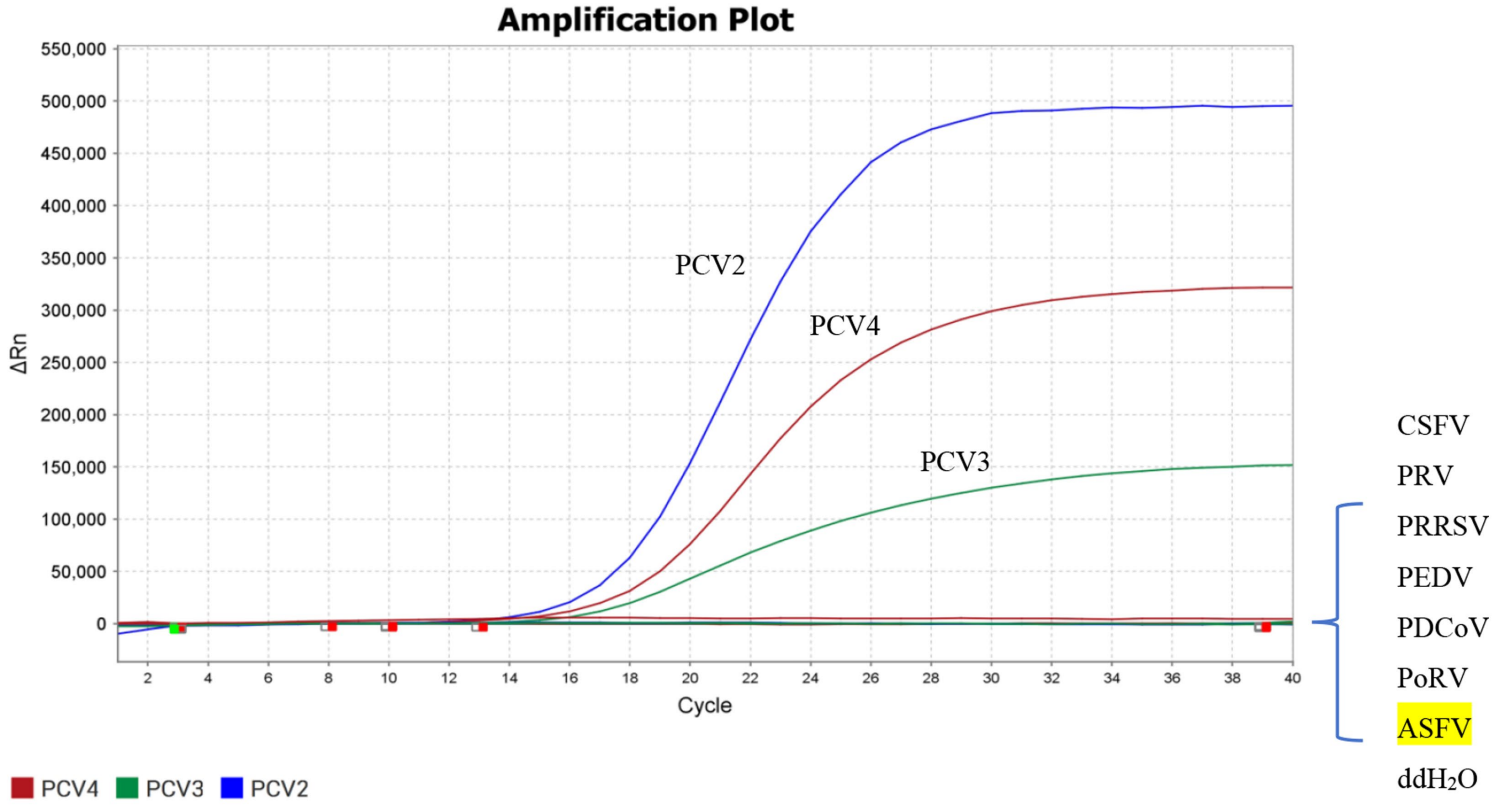
Specificity of the triplex real-time PCR assay. FAM, ROX, and HEX fluorescent signals were detected by the triplex real-time PCR assay, and the constructed plasmid was used as a positive control. No fluorescent signal was observed for any other viruses.

DNA samples of PPV, ASFV and PRV, as well as cDNA samples like PRRSV, CSFV, PEDV, PDCoV, PoRV were used as templates to evaluate the specificity of the triplex real-time PCR established in this study. As shown by Figure 2, only the FAM, ROX and HEX fluorescence signals related to PCV2, PCV3, and PCV4 could be specifically detected, while no fluorescence signals of other irrelevant pathogens could be detected. All the above results indicated that the established triplex real-time PCR has a high level of specificity.

### 3.4 Sensitivity of the triplex real-time PCR

**Figure 3:**
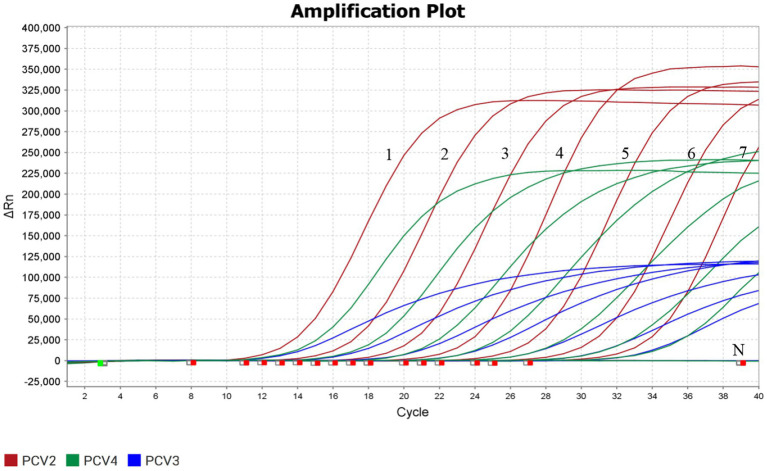
Sensitivity detection of the triplex real-time PCR assay. Labels 1–7 represented 10-fold serial diluted concentrations of standard plasmids (4.8 × 10^7^, 4.8 × 10^6^, 4.8 × 10^5^, 4.8 × 10^4^, 4.8 × 10^3^, 4.8 × 10^2^, 4.8 × 10^1^ copies/μL, respectively). The detection of PCV2, PCV3, and PCV4 were shown by red lines, blue lines and green lines, respectively.

The sensitivity of the triplex real-time PCR was determined using the plasmid with a 10-fold serial dilution (4.8 × 10^7^–4.8 × 10^1^copies/μL). ddH_2_O used as a negative control. The results showed that the limit of detection (LOD) of the triplex real-time PCR assay for detecting PCV2, PCV3, and PCV4 reached 4.8 × 10^1^ copies/μL, possessing high level of sensitivity (Figure 3).

### 3.5 Repeatability and reproducibility of the triplex real-time PCR

The repeatability and reproducibility of the triplex real-time PCR assay were evaluated by using different concentrations of standard plasmids (4.8 × 10^3^, 4.8 × 10^5^, 4.8 × 10^7^ copies/μL), and the results showed that the intra-batch and inter-batch CVs of Ct values ranged from 0.12 to 1.83% and 0.23 to 1.81%, respectively. The coefficients of variation were all less than 2%. The results showed that the triplex real-time PCR had satisfactory repeatability and reproducibility (Table 2).

**Table 2:** Intra-reproductivity and inter-reproductivity of the triplex real-time PCR assay.

Standard plasmid	Target	Intra-reproductivity (Ct value)	SD	Coefficients of variation (%)	Inter-reproductivity (Ct value)	SD	Coefficients of variation (%)
4.8 × 10^7^ copies/μL	PCV2	14.59	0.25	1.77	14.28	0.25	1.81
	PCV3	13.20	0.17	1.31	13.22	0.15	1.20
	PCV4	14.08	0.25	1.83	14.46	0.05	0.35
4.8 × 10^5^ copies/μL	PCV2	21.14	0.06	0.32	21.30	0.08	0.40
	PCV3	19.67	0.08	0.42	20.33	0.06	0.31
	PCV4	20.65	0.10	0.49	21.63	0.05	0.23
4.8 × 10^3^ copies/μL	PCV2	27.92	0.03	0.12	28.31	0.29	1.03
	PCV3	26.416	0.04	0.18	27.54	0.33	1.21
	PCV4	27.67	0.06	0.23	29.26	0.38	1.32

### 3.6 Comparison of the triplex real-time PCR with the commercial single-plex commercial real-time PCR kit

Fifteen DNA samples were used to compare the established triplex real-time PCR with PCV2 or PCV3 commercial single-plex real-time PCR kits. As shown in Table 3, the results of established triplex real-time PCR assay were completely consistent with commercial real-time PCR detection kits.

**Table 3:** The detection results of 15 DNA samples using the commercial single-plex real-time PCR detection kits and the established real-time PCR assay in this study.

Samples	Commercial real-time PCR detection kit		Established real-time PCR assay	
	PCV2	PCV3	PCV2	PCV3
1	+	−	+	−
2	+	−	+	−
3	+	−	+	−
4	+	−	+	−
5	+	−	+	−
6	−	+	−	+
7	−	+	−	+
8	−	+	−	+
9	−	+	−	+
10	−	+	−	+
11	−	−	−	−
12	−	−	−	−
13	−	−	−	−
14	−	−	−	−
15	−	−	−	−

### 3.7 Clinical applications of the triplex real-time PCR

**Figure 4:**
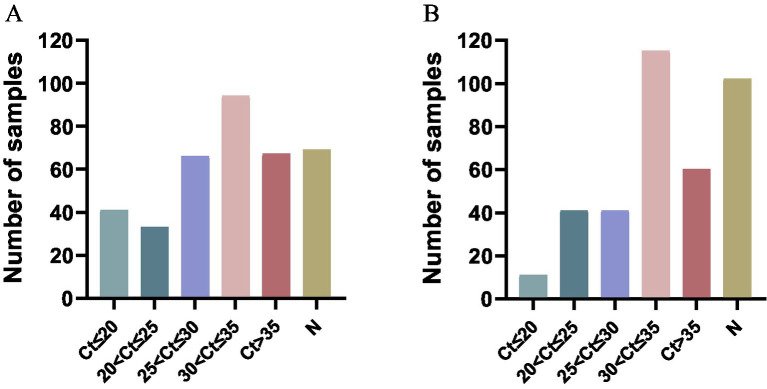
Cycle threshold (Ct) distribution of different porcine circovirus. **(A)** The proportions of PCV2 positive samples in different Ct value intervals. **(B)** The proportions of PCV3 positive samples in different Ct value intervals. “N” represents the number of negative samples.

A total of 370 clinical samples, which were collected from the north Anhui province of China, were used to investigate the prevalence of PCV2, PCV3, and PCV4 using the established triplex real-time PCR assay. The positive rates of PCV2 and PCV3 detected by the triplex real-time PCR assay were 81.35% (301/370) and 72.43% (268/370), respectively. It was found that 38.37% (142/370) of samples were co-infected with PCV2 and PCV3. PCV4 was not detected in the above samples (Table 4). Besides, as shown in Figure 4, The proportions of PCV2 positive samples in different Ct value intervals (Ct value ≤20, 20 < Ct value ≤25, 25 < Ct value ≤30, 30 < Ct value ≤35, 35 < Ct value and negative samples), which were 11.08, 8.91, 17.83, 25.41, 18.11 and 18.64%, respectively The proportions of PCV3 positive samples in different Ct value intervals were 2.97, 11.08, 11.08, 31.08, 16.21 and 27.56%, respectively. The results indicated that both the PCV2 and PCV3 were prevalently distributed in Anhui province.

**Table 4:** The detection results of 370 clinical samples.

PCV2+	PCV3+	PCV2 + PCV3+	PCV4+
301/370	268/370	142/370	0/370

## 4 Discussion

Porcine circovirus type 1 (PCV1) was first discovered by German scientist Tischer in 1974 within a porcine kidney cell line (PK-15 cells) (20). Remarkably, this particular virus does not induce pathogenicity in pigs. In 1991, porcine circovirus type 2 (PCV2) was isolated from the pathogen associated with postweaning multisystemic wasting syndrome (PMWS) by Canadian scholars and has since spread globally (21, 22). Porcine circovirus type 3 (PCV3) was first reported by American scholars in 2016 (23). PCV3 exhibits a relatively high detection rate in cases such as reproductive disorders, porcine dermatitis and nephropathy syndrome, and diarrhea (24). Porcine circovirus type 4 (PCV4) was first detected from a severe diseased pig in Hunan in 2019 (25). At present, PCV2 is the primary concern in clinical practice. The new-emerging PCV3 and PCV4 have drawn more attention from pig industry due to their economic losses. Since the pathogenicity of PCV1 is controversial (26, 27), and it is less harmless than PCV2, PCV3, and PCV4. Therefore, the established triplex assay in this study excluded PCV1. These three pathogens all have similar symptomatic manifestations and are difficult to distinguish, thereby imposing substantial economic losses to the global pig industry. In recent years, the prevalence of these viruses has shown an upward trend across different provinces in China. After being infected by PCV2, pigs will experience immunosuppression, which subsequently gives rise to mixed infections with other pathogens. Therefore, rapid differential diagnosis and the assessment of the severity of the disease represent the foremost and essential steps in formulating an effective disease control strategy. The purpose of this study is to develop a rapid, reliable and high-throughput triplex real-time PCR assay for detecting of PCV2, PCV3, and PCV4. Compared with other assays, the real-time PCR assay has the advantages of high sensitivity and the capacity to observe the viral load in real time. Therefore, in this study, a triplex real-time PCR assay that can simultaneously detect PCV2, PCV3, and PCV4, was established. It can promptly conduct differential diagnosis of the three pathogens in the same reaction system. This assay has satisfactory specificity, sensitivity, repeatability and reproducibility. To evaluate the accuracy of the established triplex real-time PCR assay, 15 DNA samples were used to compare the detection results between the established triplex real-time PCR assay and the commercial single-plex real-time PCR kits. The results of the two detection methods were completely consistent.

To investigate the prevalence of PCV in the northern Anhui of China, 370 viral nucleic acid samples were extracted and then detected using the established assay. The results showed that the positive rate of PCV2 was 81.35% (301/370), the positive rate of PCV3 was 72.43% (268/370), and no PCV4 was detected. The co-infection rate of PCV2 and PCV3 was 38.37% (142/370). The clinical samples were collected from northern Anhui Province. Therefore, the results of this study only represent the prevalence of PCVs in northern Anhui Province, which limits the representativeness in a wider area. The pig-raising environments, immunization statuses and virus prevalence vary in different regions, and these factors may lead to different virus infection situations. Guilherme Cezar et al. (28) conducted a study on 14,915 samples from 6 laboratories in the United States. Their research revealed a significant correlation between the PCR Ct value and the diagnosis of PCV2/PCV3 diseases. As the PCR Ct value decreased, the likelihood of clinical diseases increased. This was achieved through the analysis of 14,915 samples with PCR detection results and samples based on tissue assessment results. When the Ct value of PCV2 PCR was lower than 22.4, there was an 81.63% accuracy in determining that the case was PCVAD. For PCV3, when the Ct value was lower than 26.7, 70.99% of the samples were evaluated as PCVAD through tissue assessment. The Ct values obtained by real-time PCR indirectly reflect the viral load present in the samples, the magnitude of the Ct value is inversely proportional to the viral load, the lower the Ct value, the higher the viral load, and the greater the likelihood of infection. In this study, the proportion of PCV2-positive samples with a Ct value ≤25 was 19.99%, and the proportion of PCV3-positive samples with a Ct value ≤25 was 14.05%. It indicates that the prevalence of PCV2 and PCV3 in the northern Anhui is relatively high, it is necessary to take measures for prevention and control.

In recent years, it is reported that PCVAD symptoms are often accompanied by co-infection of PCV2 and PCV3 (29), the co-infection rate of the two in swine farms is generally 27.6–39.39% (14, 30, 31), it is consistent with the co-infection rate of PCV2 and PCV3 in Anhui province in this study. Keli et al. (12) detected 286 samples from the central China region and found that the positive rates of the three genotypes, PCV1, PCV2 and PCV3, were 52.4% (150/286), 61.2% (175/286) and 45.1% (129/286), respectively. Yang et al. (32) detected 257 clinical samples in the southwest of China, the positive rates of PCV2 and PCV3 were 26.46% (68/257) and 33.46% (86/257). Li (33) collected a total of 15,130 samples from eight province, the positive rate of PCV2 ranged from 8.31 to 42.36%. The above data indicate that PCV2 and PCV3 are more widely distributed and transmitted in most provinces in China. Since 2019, PCV4 has been reported in several provinces in China, including Shandong, Henan, Shanxi, Jiangsu, and Guangxi (25, 34–36). PCV4 was not detected in this study. Possible reasons include not only insufficient sample quantity. It is possible that PCV4 was not prevalent locally. Additionally, local breeding conditions, immunization status can also affect the infection and detection of PCV4. To prevent and control PCV4, continuous monitoring of pigs in Anhui province is necessary.

In conclusion, this study has successfully established a triplex real-time PCR assay for PCV2, PCV3, and PCV4. This assay exhibits satisfactory specificity, sensitivity, repeatability, and reproducibility and can be well applied to the detection of clinical samples, providing a reliable tool for the rapid identification of PCV2, PCV3, and PCV4.

## Data Availability

The raw data supporting the conclusions of this article will be made available by the authors, without undue reservation.
