# Supplementary material for: Establishment and application of a triplex real-time PCR assay for detection of porcine circoviruses
Source: Front Vet Sci. 2025 Mar 3;12:1558389. doi: 10.3389/fvets.2025.1558389 (PMC11911328; doi:10.3389/fvets.2025.1558389)
Supplement: Supplementary file 1 [file Table_1.DOCX]

Supplementary data

Figure S1. Design of primers and probes for PCV2, PCV3, and PCV4. (A) Design of primers and probes for PCV2; (B) Design of primers and probes for PCV3; (C) Design of primers and probes for PCV4.


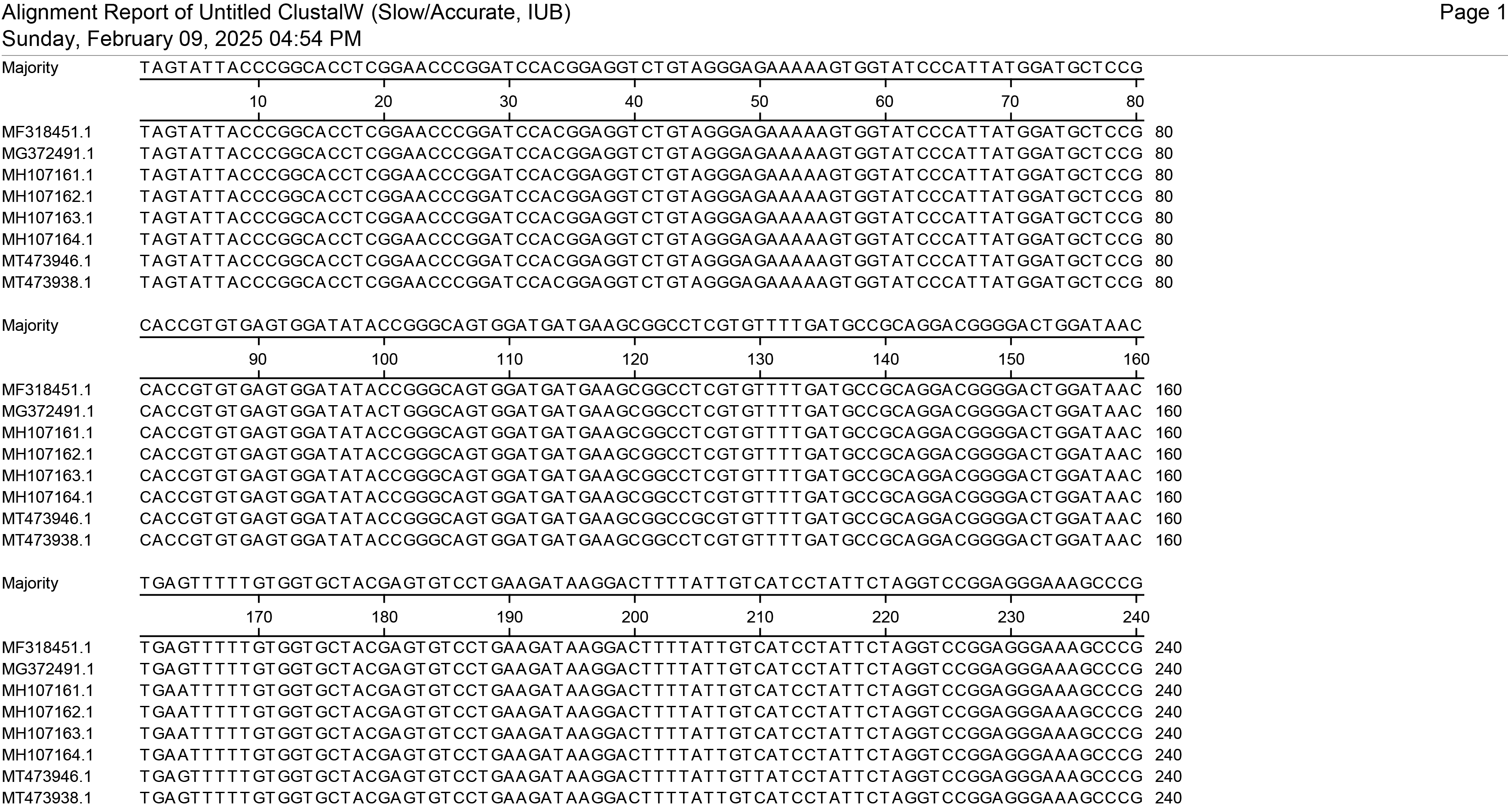

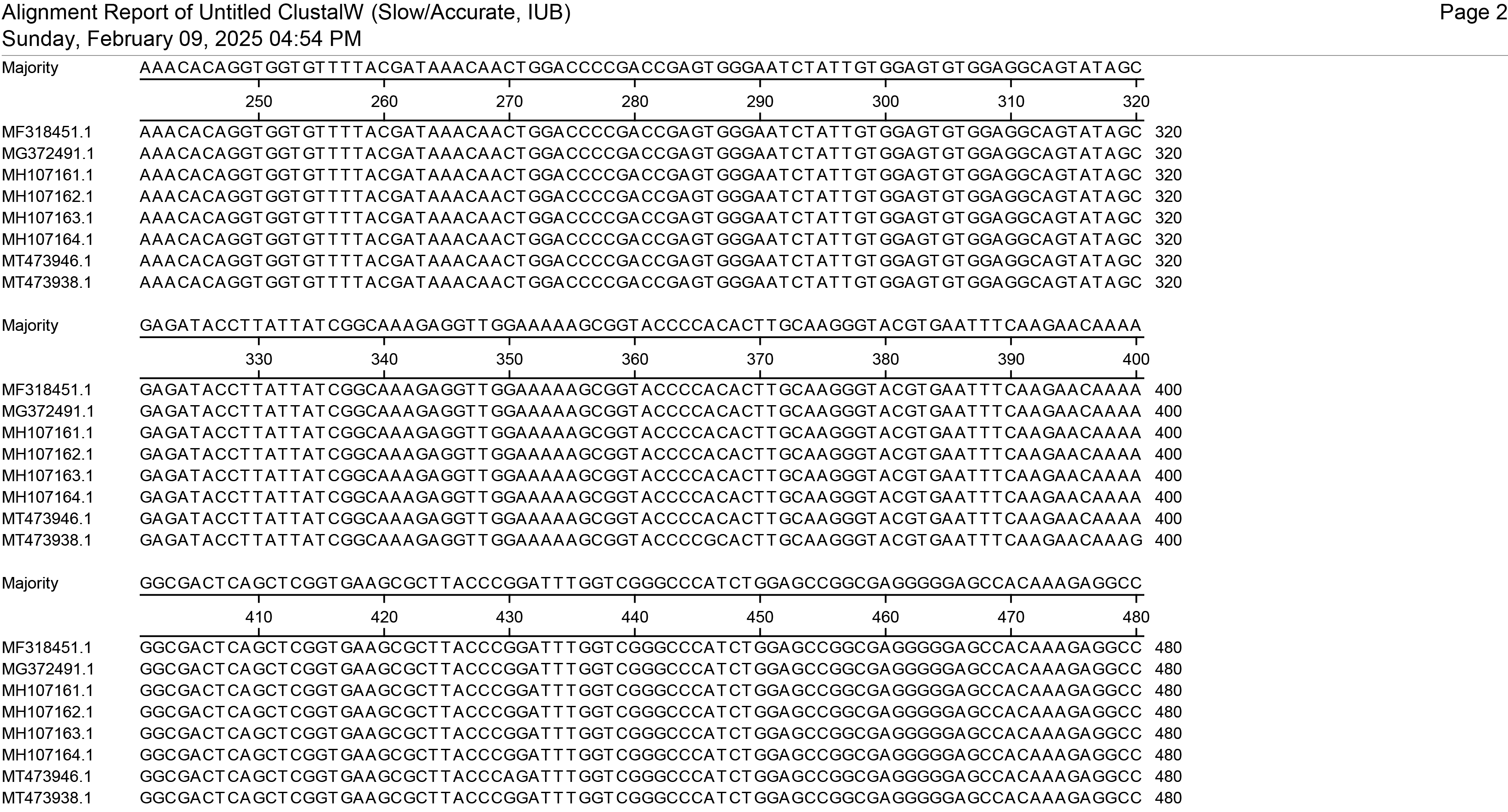


(B)

Forword primer

ROX

MGB

Reverse Primer


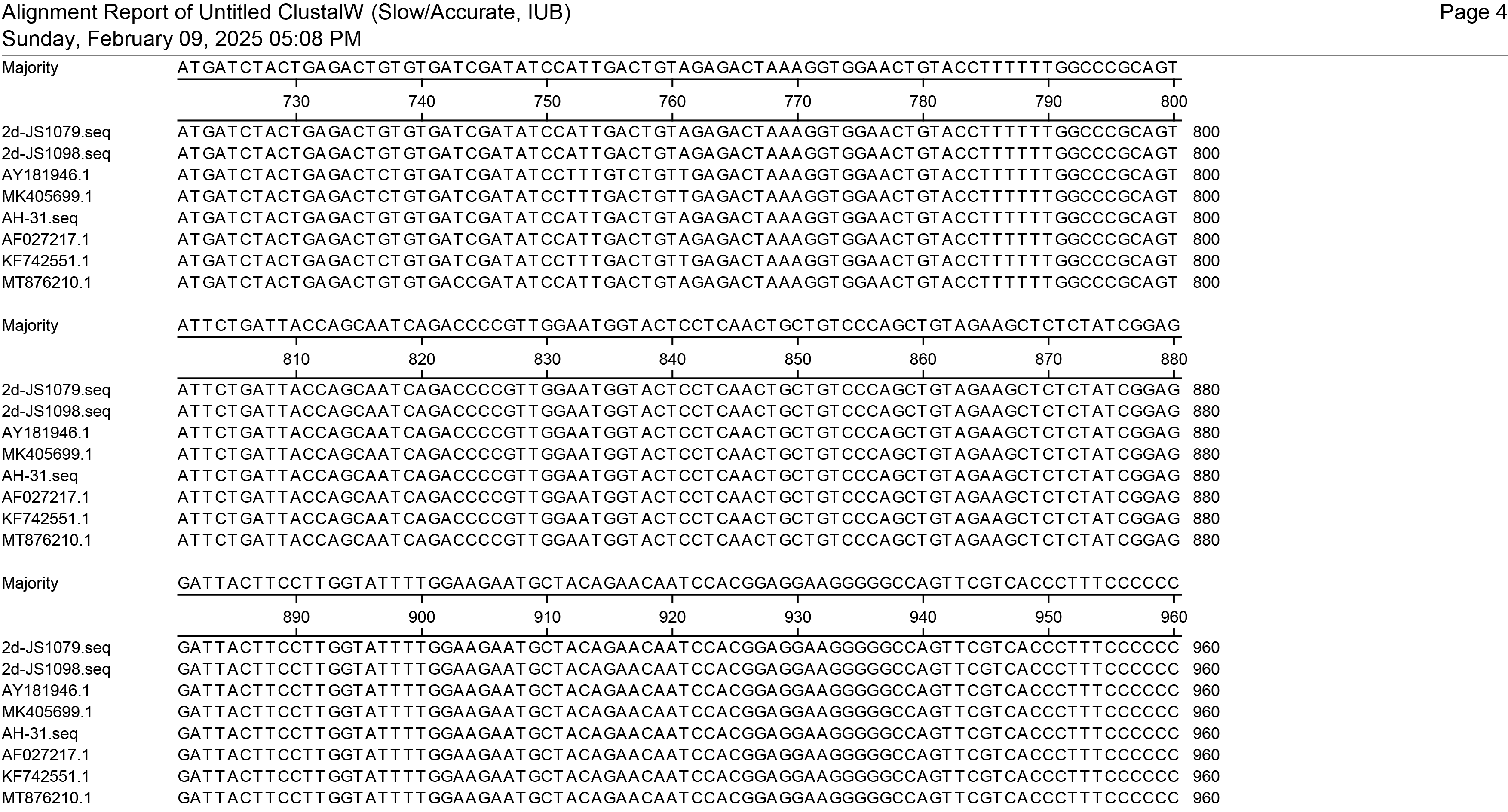


(A)

FAM

MGB

Forword primer

Reverse Primer


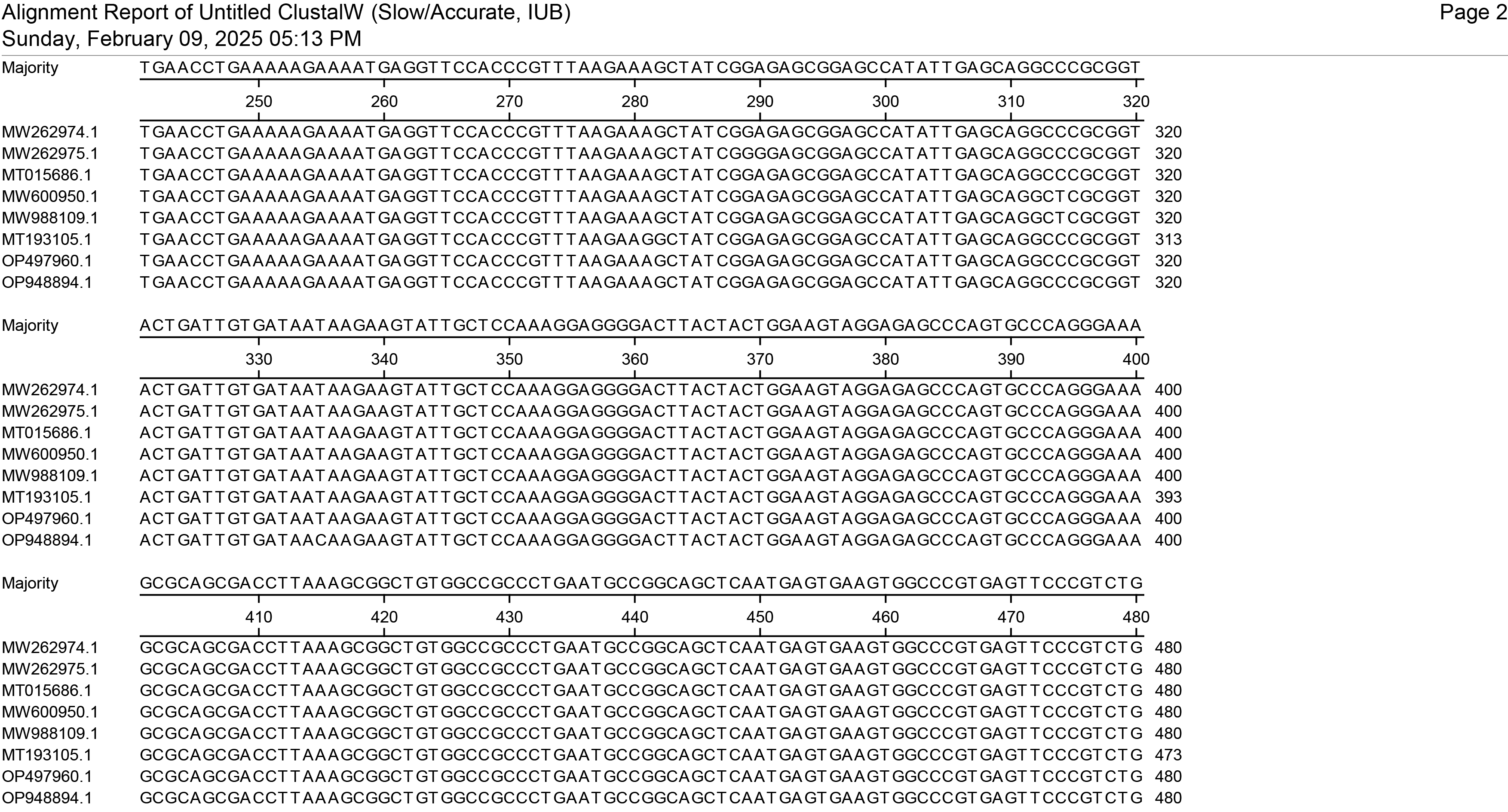


(C)

Forword primer


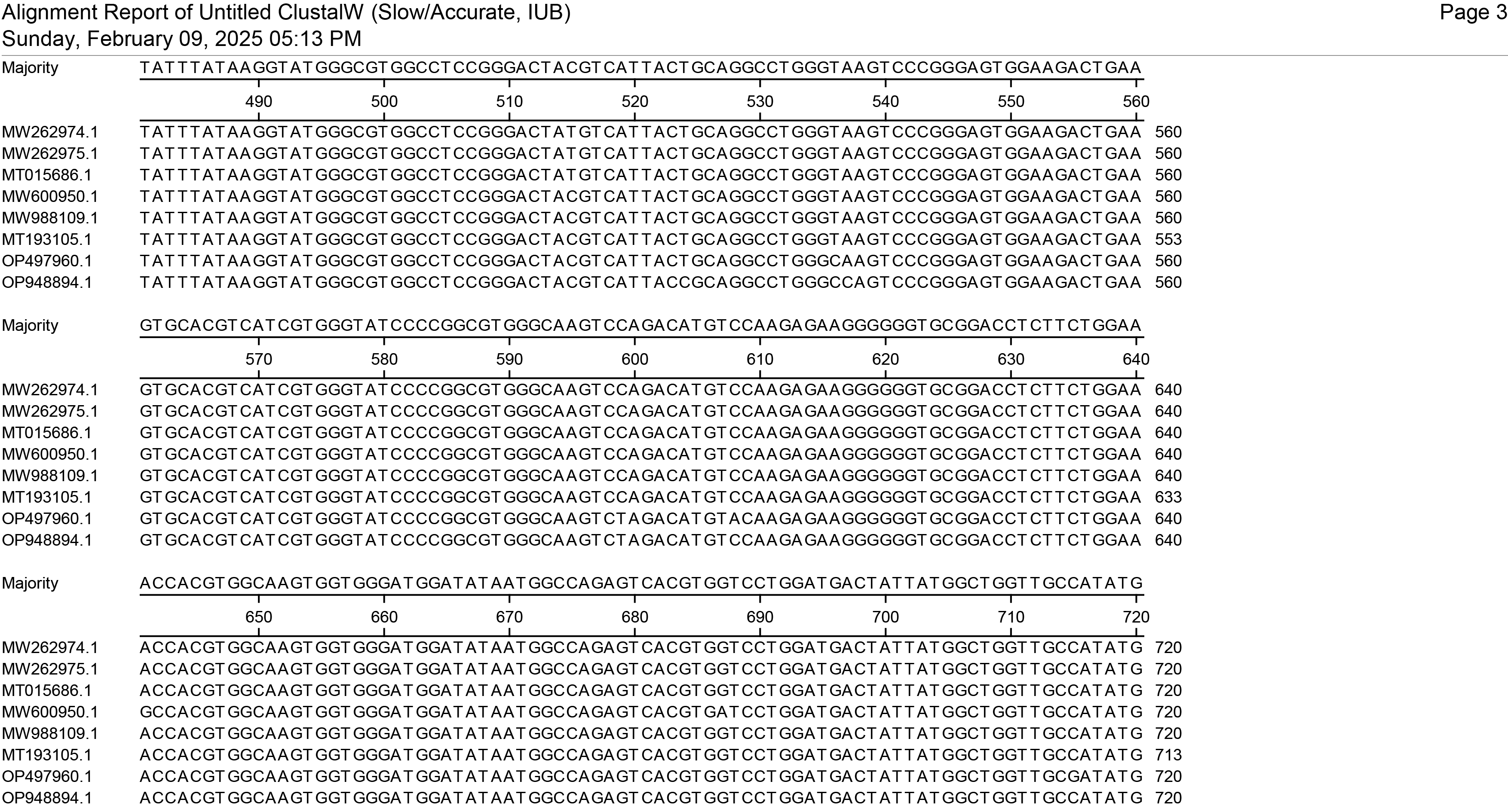


HEX

MGB

Reverse Primer
